# Big data analysis for Covid-19 in hospital information systems

**DOI:** 10.1371/journal.pone.0294481

**Published:** 2024-05-22

**Authors:** Xinpa Ying, Haiyang Peng, Jun Xie

**Affiliations:** Hospital of Chengdu University of TCM, Chengdu, Sichuan, China; University of Engineering and Applied Sciences, PAKISTAN

## Abstract

The COVID-19 pandemic has triggered a global public health crisis, affecting hundreds of countries. With the increasing number of infected cases, developing automated COVID-19 identification tools based on CT images can effectively assist clinical diagnosis and reduce the tedious workload of image interpretation. To expand the dataset for machine learning methods, it is necessary to aggregate cases from different medical systems to learn robust and generalizable models. This paper proposes a novel deep learning joint framework that can effectively handle heterogeneous datasets with distribution discrepancies for accurate COVID-19 identification. We address the cross-site domain shift by redesigning the COVID-Net’s network architecture and learning strategy, and independent feature normalization in latent space to improve prediction accuracy and learning efficiency. Additionally, we propose using a contrastive training objective to enhance the domain invariance of semantic embeddings and boost classification performance on each dataset. We develop and evaluate our method with two large-scale public COVID-19 diagnosis datasets containing CT images. Extensive experiments show that our method consistently improves the performance both datasets, outperforming the original COVID-Net trained on each dataset by 13.27% and 15.15% in AUC respectively, also exceeding existing state-of-the-art multi-site learning methods.

## Introduction

The global public health crisis caused by COVID-19 continues to spread. Medical imaging, particularly computed tomography (CT), plays a critical role in the clinical diagnosis [1] and monitoring of patients with COVID-19. However, the increasing number of COVID-19 suspicious cases has overloaded public health services, revealing a shortage of trained radiologists. Therefore, effective computational methods are needed to automate COVID-19 CT image analysis, improve diagnosis outcomes and patient management, and reduce the tedious workload of clinicians on image interpretation, allowing them to dedicate more time to urgent matters on the frontline.

**Fig 1:**
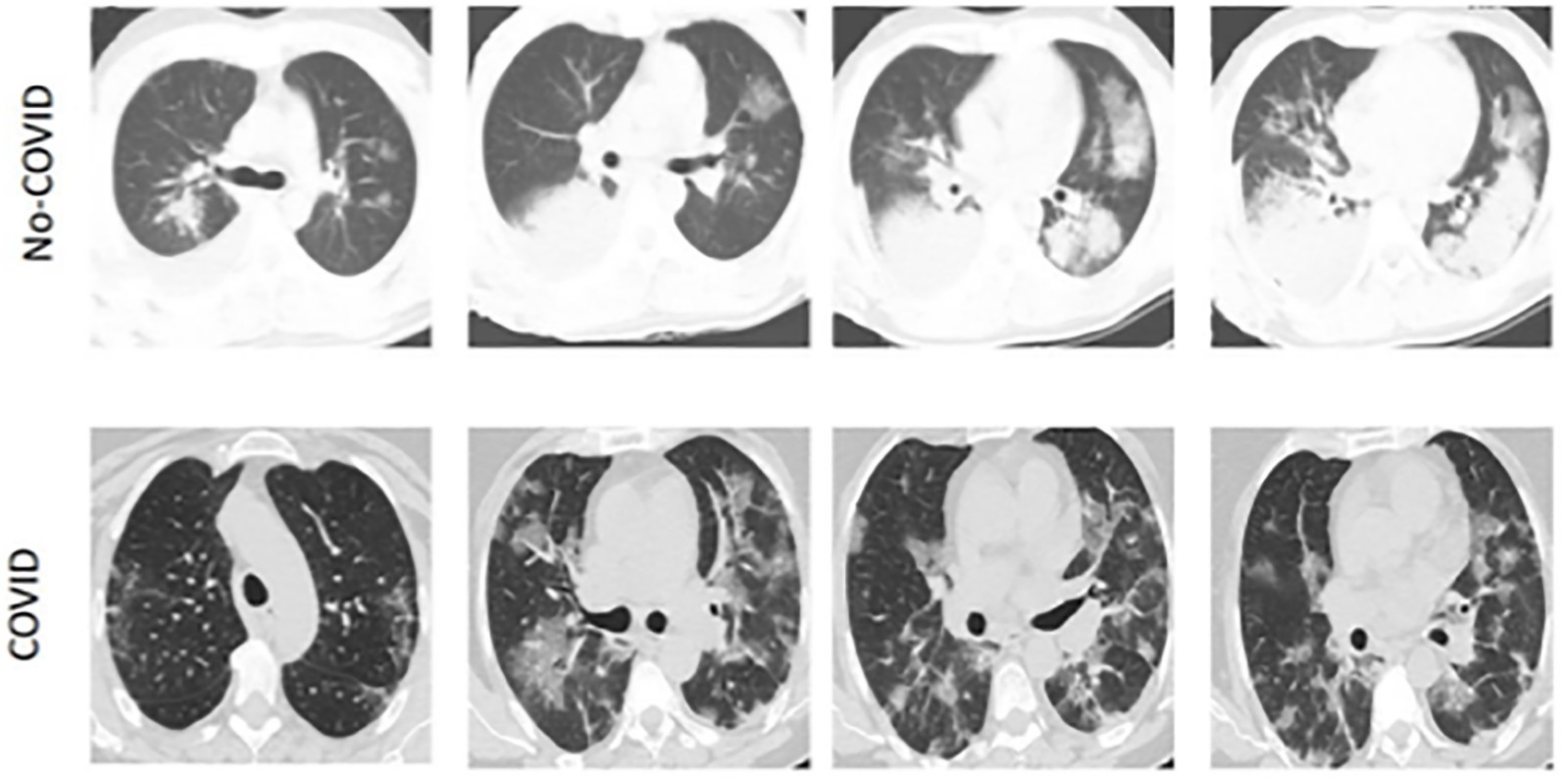
The appearance and contrast of CT images from COVID-19 patients vary significantly across two different clinical centers, indicating data heterogeneity.

In the era of big data, many data-driven methods have developed rapidly, with high accuracy typically attributed to the collection of large-scale datasets [2–4]. However, collecting these datasets in practice is often difficult. To address the issue of insufficient single-site data, it is necessary to aggregate CT data from different hospitals to establish a cross-hospital learning scheme. For example, Di et al. proposed a hypergraph model for multi-hospital pneumonia data to achieve rapid identification of COVID-19 cases [5] shown in Fig 1. Wang et al. also collected datasets from different hospitals to build an accurate X-ray image classifier [6]. However, all of these works have neglected the heterogeneity of data across different hospitals, such as imaging protocols and differences in CT equipment. CT slices from different datasets exhibit different image contrasts, which may weaken a model’s generalization ability. Previous works [7–9] have found that simple improvements on such heterogeneous data often bring limited improvement, or even worse performance than training on a single dataset.

Our research aims to address the practical challenge of accurately identifying COVID-19 CT images by using an effective joint learning framework to handle distribution heterogeneity. We not only redesigned the latest COVID-Net [6] network architecture and learning strategy, but also conducted effective joint learning on a new backbone network, fully leveraging the advantages of multiple datasets. We also used domain-specific batch normalization layers to estimate internal feature statistics for each site separately and normalize the features. Our method aims to explicitly adjust the category sensitivity and domain invariance of the latent semantic feature space and proposes a contrastive learning objective. We evaluated our method on two public COVID-19 CT classification datasets, and extensive experiments showed that our joint learning framework outperforms single-site training models, simple joint learning, and existing state-of-the-art multi-site learning methods on both datasets. Our method effectively combines multiple data sources by proposing a joint learning framework, improving the quality of COVID-19 CT image recognition, and making a significant contribution to the field. Our research not only improves the accuracy of COVID-19 CT image recognition but also has the potential to help doctors diagnose COVID-19 more accurately in practice, providing support for epidemic prevention and control.

- Our focus has been on enhancing the computational efficiency and prediction accuracy of the COVID-Net [6] specifically for COVID-19 CT images. To achieve this, we have revamped the original COVID-Net by revising its network architecture and learning strategies, which were originally developed for X-Ray.
- Our proposed joint learning framework improves COVID-19 diagnosis by effectively leveraging heterogeneous datasets. We address the issue of inter-site data discrepancy by performing separate feature normalization and proposing a contrastive objective to explicitly enhance the robustness of semantic representations.
- We extensively evaluated our method on two public datasets, using rigorous experimentation. The results consistently demonstrate significant improvement in classification performance on both datasets.

## Related works

### COVID-19 screening

A significant amount of research has been conducted with great speed and intensity to develop deep learning methods for responding to the global COVID-19 pandemic [10]. In this paper, we provide a brief overview of deep learning methods for image-level classification in diagnosis, which are closely related to the focus of our study.

Initially, Khan et al. [11] aimed to develop a large-scale screening model using chest CT images to distinguish COVID-19 pneumonia [11–14], Influenza-A viral pneumonia, and healthy cases. To achieve this, they employed ResNet18 with both location-attention mechanism and channel attention mechanism. Several follow-up methods based on transfer learning were proposed later, with most of them utilizing well-established network architectures such as VGG [15], ResNet [16–18], and DenseNet [19].

During this period, researchers proposed several new network architectures. COVID-Net, for example, was specifically designed for COVID-19 recognition and achieved high accuracy for image-level diagnosis based on chest X-ray (CXR) images [6]. Alqahtani et al. [20] using edges exploitation operation in an optimized structure with the convolutional operator facilitates learning edges-related features of infection patterns in chest X-ray images. Khan et al. [21] proposes a new deep CNN based technique for COVID-19 classification in X-ray images which systematically employs Region and Edge-based operations along with convolution operations. Zahoor et al. [22] propose a novel two-pahse deep learning-based framework to detect and categorize brain tumors in magnetic resonance images. Khan et al. [23] propose two new deep learning frameworks including Deep Hybrid Learning and Deep Boosted Hybrid Learning for effective COVID-19 detection in X-ray dataset. Meanwhile, Khan et al. [24] propose a novel semantic segmentation model CoV-RASeg which systematically uses average and max pooling operations in the encoder and decoder blocks. Khan et al. [25] also provide a survey of deep learning techniques for the analysis of COVID-19 and their usability for detecting omicron.

Javaheri et al. also designed CovidCTNet for detecting COVID-19 infections based on CXR images [2]. Another framework based on Capsule Network aimed to handle smaller datasets more effectively [26]. Gozes et al. proposed a system that utilized robust 2D and 3D deep learning models by modifying and adapting existing AI models and combining them with domain-specific clinical understanding [27]. Tang et al. explored severity-related features for automated severity assessment of COVID-19 based on chest CT images [28]. Rahimzadeh et al. concatenated features from Xception and ResNet50V2 networks to develop a neural network that achieved improved recognition performance [29].

### Contrastive learning

Contrastive learning is a technique that aims to learn representations by contrasting positive and negative sample pairs [12, 13, 30]. It has been widely used in recent works on instance-level discrimination in self-supervised learning. This approach aims to learn representations by imposing transformation invariances in the latent space. In these works, the exemplar CNN represented each instance as a vector and trained a network to recognize each instance. However, one important issue in contrastive learning is negative sampling. To address this, Wu et al. proposed a memory bank to store instance vectors [31]. He et al. proposed the momentum contrast for visual representational learning [32]. More recently, Chen et al. proposed SimCLR, a method for generating augmentation-invariant embeddings for input images [33]. These works have achieved significant empirical successes in self-supervised representation learning.

Several research works have explored the potential of contrastive learning for COVID-19 screening. For instance, He et al. integrated contrastive learning and transfer learning to pre-train classification networks for COVID-19 diagnosis. Li et al. proposed a contrastive multi-task CNN that can improve generalization on unseen CT or X-ray samples for COVID-19 diagnosis [34].

In our study, we extensively utilize contrastive learning to obtain highly discriminative representations. In addition to conventional contrastive learning, we incorporate category information to better explore intra-class similarity and inter-class difference. Specifically, we adopt it as an auxiliary learning task that effectively enhances the classification performance of COVID-19.

## Methods

In this section, we first discussed our process of designing COVID-Net. We redesign the original COVID-Net as backbone and performs seprate feature normalization to tackle the statoistical problem.

Then, we introduced our deep learning scheme, including addressing the issue of different data quality from different hospitals and feature normalization. Additionally, we introduced contrastive learning to learn domain-invariant features, resulting in improved classification performance of our model.

### COVID-Net redesign for improved CT classification

Our model is based on COVID-Net [6], a deep learning architecture designed specifically for COVID-19 chest X-ray images that has outperformed several popular classification networks pretrained on ImageNet. Our network consists of two modules: a light upper branch with four convolutional layers and a lower branch composed of dense convolutional blocks for deep feature extraction. Long-range multi-level feature fusion is achieved through skip connections between the two branches. However, COVID-Net [6] was optimized for relatively coarse lesions in chest X-ray images and may not be ideal for CT images where the lesion pattern is clearer and provides richer information for the model to learn. Therefore, our goal is to build upon the strengths of this backbone while improving learning efficiency and classification accuracy from two complementary angles.

#### Network architecture redesign

The original COVID-Net [6] has a limitation in that it lacks internal feature normalization layers, which results in significant differences in the learned representations across different layers and branches. This feature variance is further amplified in CT images, which contain more complex patterns, and can potentially affect the training process and prediction accuracy if not appropriately addressed. To overcome this issue, we added batch normalization [35] (BN) layers to specific components of the network, reducing internal covariate shift, enhancing feature discrimination ability, and speeding up convergence rate. However, adding BN layers to every convolution layer is not necessarily beneficial. The computation blocks in the lower branch contain densely connected short-range connections, and adding BN layers to them would significantly increase the parameter scale and decrease training speed. Therefore, we added a BN layer after each convolutional layer in the upper branch and one for the initial convolutional layer to balance computational efficiency and stable representation.

Formally, the batch normalization (BN) operation obtains normalized features $x=\{x_{1},\ldots ,x_{M}\}$ by applying affine transformation to the whitened feature maps along each channel *iin*1, *dots*, *M*, given *M*-channel feature maps $y=\{y_{1},\ldots ,y_{M}\}$ of a certain layer:

$$\begin{array}{c} y_{i}=\gamma \hat{x_{i}}+\beta ,\;\text{where}\;\hat{x_{i}}=\frac{x_{i}-\mu_{i}}{\sqrt{\sigma_{i}^{2}+\epsilon }}, \end{array}$$ (1)

**Fig 2:**
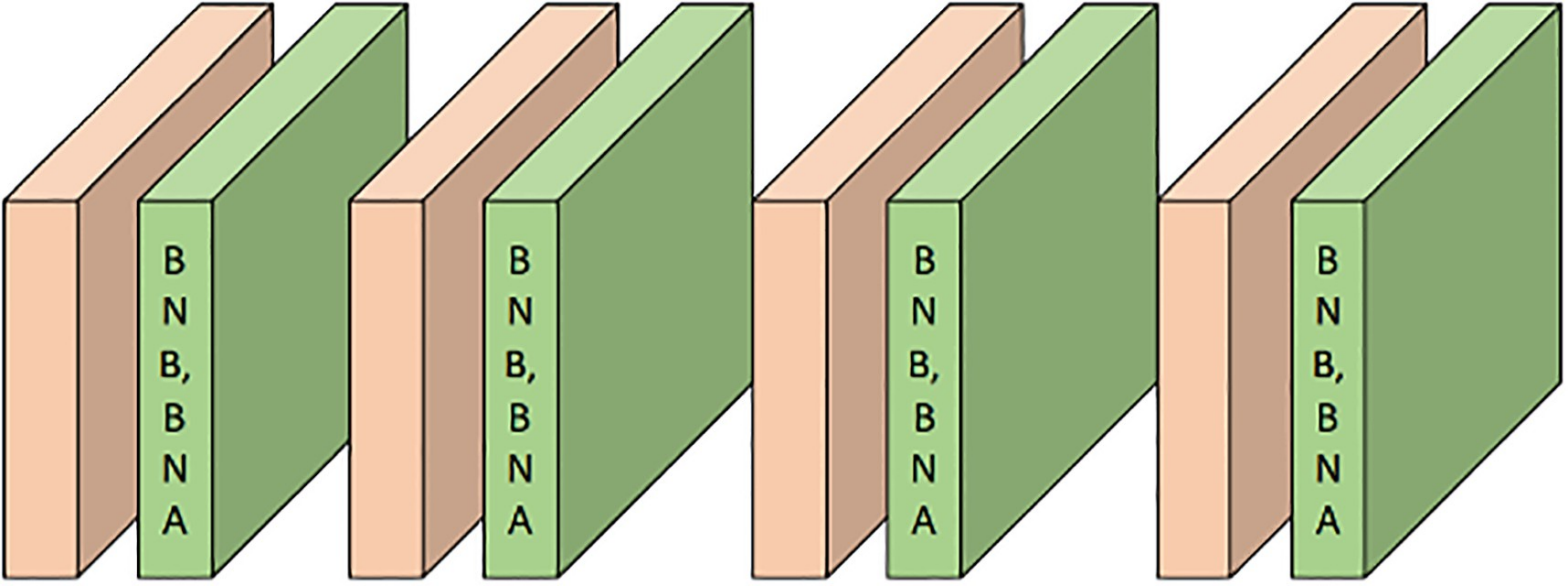
The COVID-Net redesign for improved CT classification.

In the above equation, *μ*_*i*_ and $\sigma_{i}^{2}$ represent the mean and variance of feature *x*_*i*_, respectively, the framework is shown in in Fig 2; The BN layer collects moving average values of *γ* and *β* during training to capture global data statistics and employs these estimated values for feature normalization during the testing phase.

### Joint learning scheme with redesigned COVID-Net

Given the limited number of COVID-19 samples from individual hospitals, it is often necessary to aggregate cases from different data sources for deep learning model development. Therefore, we propose a joint learning scheme, built upon the redesigned COVID-Net backbone, to explicitly address the data heterogeneity problem and improve diagnosis performance.

#### Separate batch normalization (SBN) at data heterogeneity

In the presence of significant data heterogeneity, simple joint training has been shown to have limitations or even lead to performance degradation [7, 14, 38]. One significant factor contributing to this is inaccurate estimation of moving average values by the BN layer during training due to statistical differences across datasets, as depicted in Fig 2. Consequently, these estimated values may not accurately represent the testing data statistics at each site, leading to degraded performance. To overcome this issue, we utilize domain-specific batch normalization (DSBN) [7, 39, 40] by assigning a BN layer to each site to explicitly address statistical discrepancies. As illustrated in Fig 2, we substitute the BN layers in the redesigned COVID-Net with DSBN layers. The DSBN layer captures domain-specific moving values that accurately represent the statistics of each site, outperforming the original BN layer.

#### Contrastive domain invariance enhancement (CDIE)

In joint learning, in addition to addressing the issue of different data distributions across sites, we also aim to obtain robust semantic information that can cluster different source domain data. This is crucial for domain adaptation, as if the model cannot cluster data of the same class in the source domain, data from multiple hospitals cannot be fully utilized. To tackle this issue, we propose utilizing contrastive learning to cluster data within the same class while separating data between different classes.

To achieve this goal, we adopt the popular contrastive learning method [41]. Given a pair of samples (*m*, *n*), we obtain their semantic feature embeddings *e*_*m*_ and *e*_*n*_, extracted through global average pooling layer with a vector dimension of 8096. We found that directly applying regularization to the original features hindered model convergence. Therefore, we introduce an embedding network *H*_*ϕ*_ to project the embeddings into a lower-dimensional space. The final contrastive learning formula we obtained is as follows:

$$\begin{array}{c} sim\left(m,n\right)=\frac{H_{\phi }\left(e_{m}\right)\cdot H_{\phi }\left(e_{n}\right)}{\|H_{\phi }\left(e_{m}\right){\|}_{2}\cdot \|H_{\phi }\left(e_{n}\right){\|}_{2}}. \end{array}$$ (2)

Positive pairs (*m*, *n*) refer to samples that belong to the same class, while negative pairs refer to samples that belong to different classes. In each iteration, we randomly sample a minibatch of *K* examples from the two sites. The contrastive loss for each positive pair (*m*, *n*) within the minibatch is defined as follows:

$$\begin{array}{c} \ell_{contrastive}\left(m,n\right)=-log\frac{exp(sim(m,n)/\tau )}{\sum_{k=1}^{K}F\left(m,k\right)\cdot exp\left(sim\left(m,k\right)/\tau \right)}, \end{array}$$ (3)

In this context, F(m,k) takes a value of 1 and 0 for positive and negative samples, respectively, where *τ* represents a temperature parameter. The loss function is ultimately calculated on a mini-batch of positive samples, considering both (*m*, *n*) and (*n*, *m*). Through this contrastive learning approach, the model can learn domain-invariant features that bring samples from the same class together and push those from different classes apart.

### Overall training objective and technical details

The overall training objective $L_{overall}$ is composed of two terms: the cross-entropy loss $L_{ce}$ to assess classification error and the contrastive loss $L_{con}$ to regularize the latent space. The objective is defined as:

$$\begin{array}{c} L_{overall}=L_{ce}+\alpha \cdot L_{con}, \end{array}$$ (4)

## Experiments

### Datasets and evaluation metrics

To evaluate the deep learning framework proposed in this study, we used two public medical image datasets: *SARS-CoV-2* [42] and *COVID-CT* [43]. These datasets are currently widely used and the largest publicly available datasets. The *SARS-CoV-2* dataset (referred to as Site A) consists of 2482 CT images from 120 patients, with 1252 positive and the rest negative. The spatial sizes of these images range from 119 × 104 to 416 × 512. The emphCOVID-CT dataset (referred to as Site B) includes 349 CT images from 216 COVID-19 patients and 397 CT images from 171 non-COVID-19 patients. The resolutions of these images range from 102 × 137 to 1853 × 1485. We first resized all images to 224 × 224 and then normalized them.

We conducted a four-fold cross-validation on our experiment using two datasets citeSoares2020sars, and we adopted five metrics to provide a comprehensive evaluation of our models, including accuracy (%), F1 score (%), sensitivity (%), precision (%), and AUC (%). We also presented the mean and standard deviation of three independent runs.

### Experimental setting

**Fig 3:**
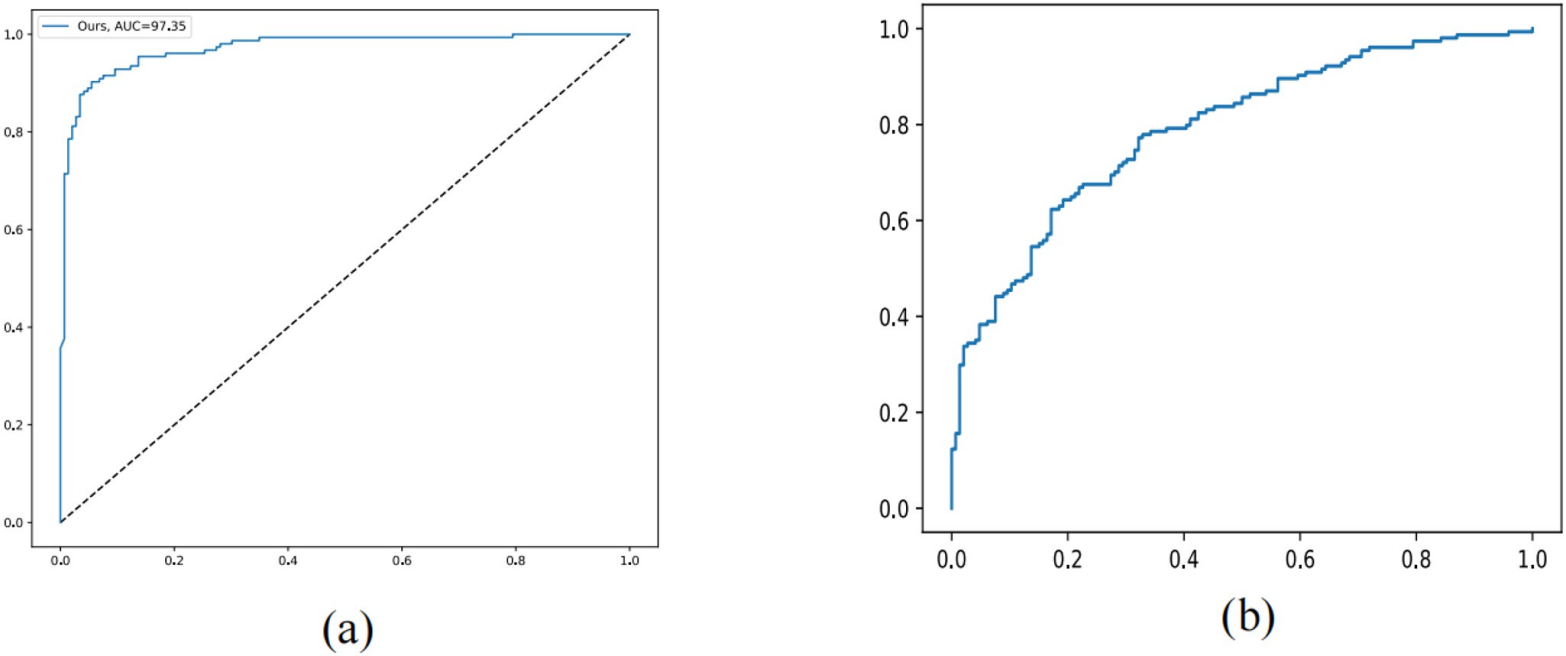
(a) ROC curves of method. (b) PR curves of our method.

We implemented the framework using Pytorch on an Nvidia Tesla V100 GPU. Both the classification model and embedding network were trained from scratch using the same Adam Optimizer. We initialized the learning rate to 1e-4 and decayed it using cosine annealing. To empirically adjust the hyper-parameters, we used grid search with a random small subset of the entire dataset and set the temperature parameter *τ* to 0.05. Fig 3 displays the ROC curves and PR curves of our approach. For our proposed method and all the comparison methods, we trained a total of 100 epochs with a batch size of 32, consisting of 16 images from each dataset.

### Effectiveness of network redesign on COVID-Net

To validate the effectiveness of our network redesign, we compared our redesigned backbone with the original COVID-Net. Two different experimental settings were used for the comparison: 1) the *Single* setting, which trains a model for each site separately, and 2) the *Joint* setting, which trains a model jointly using both datasets with naive aggregation. The results in Table 1 demonstrate that our *Redesign* model outperforms the original COVID-Net [6] in the *Single* setting on both sites by a significant margin, with consistent improvements observed across all five evaluation metrics. The *Joint* setting yielded similar observations, except for a slightly marginal improvement in precision in Site B. These findings underscore the superior representation learning ability of our redesigned backbone for COVID-19 diagnosis, indicating that our redesign strategy can effectively enhance the model’s discriminative power.

**Table 1:** Results of different methods on the two datasets for COVID-19 CT image classification (mean±std).

Methods	Site A					Site B				
	Accuracy	F1	Recall	Precision	AUC	Accuracy	F1	Recall	Precision	AUC
Single (COVID-Net [6])	77.12	76.03	70.97	80.04	84.08	63.12	61.09	57.73	64.03	71.09
Single (Redesign)	89.09	88.97	83.78	94.58	94.12	77.07	77.04	74.69	79.48	84.13
Joint (COVID-Net [6])	68.72	69.17	69.41	68.27	74.78	63.27	59.78	54.19	64.27	68.12
Joint (Redesign)	78.42	77.86	74.07	80.82	85.72	69.67	66.89	66.94	64.98	72.48
Series Adapter [36]	85.73	86.19	81.91	90.98	92.93	70.01	67.08	74.91	63.04	73.92
Parallel Adapter [37]	82.13	82.39	80.02	83.51	89.99	74.93	73.46	71.81	79.84	80.29
MS-Net [7]	87.98	88.73	84.91	93.78	94.37	76.23	76.54	74.07	79.29	82.19
SepNorm	88.76	87.88	82.97	95.46	94.57	76.89	75.02	70.34	**80.74**	83.94
+ Ours	**91.07**	**91.05**	**85.76**	**96.32**	**97.35**	**79.34**	**78.21**	**79.07**	78.39	**86.24**

#### Ablation study

We conduct an extensive ablation study to investigate the role of each component in our method in Table 2. SepNrom [44] denotes that we only use SepNorm without any other strategy. Based on SepNorm, we add a SBN (separate batch normalization) to obtain SepNorm + SBN and CDIE (contrastive domain invariance ehancement) to obtain SepNorm + CDIE. We can see that our method can improve the baseline SepNorm by a large margin. This confirm the effectiveness of the proposed method, and the flops of this paper is 1.55G.

**Table 2:** Ablation study on different components of our method on the COVID-19 images.

Methods	Accuracy	F1	Recall	Precision	AUC
SepNrom [44]	88.76	87.88	82.97	95.46	94.57
SepNorm + SBN	89.13	88.24	83.81	95.86	95.71
SepNorm + CDIE	90.23	89.74	84.28	96.01	96.34
Ours	**91.07**	**91.05**	**85.76**	**96.32**	**97.35**

#### Evaluation of domain divergence

**Fig 4:**
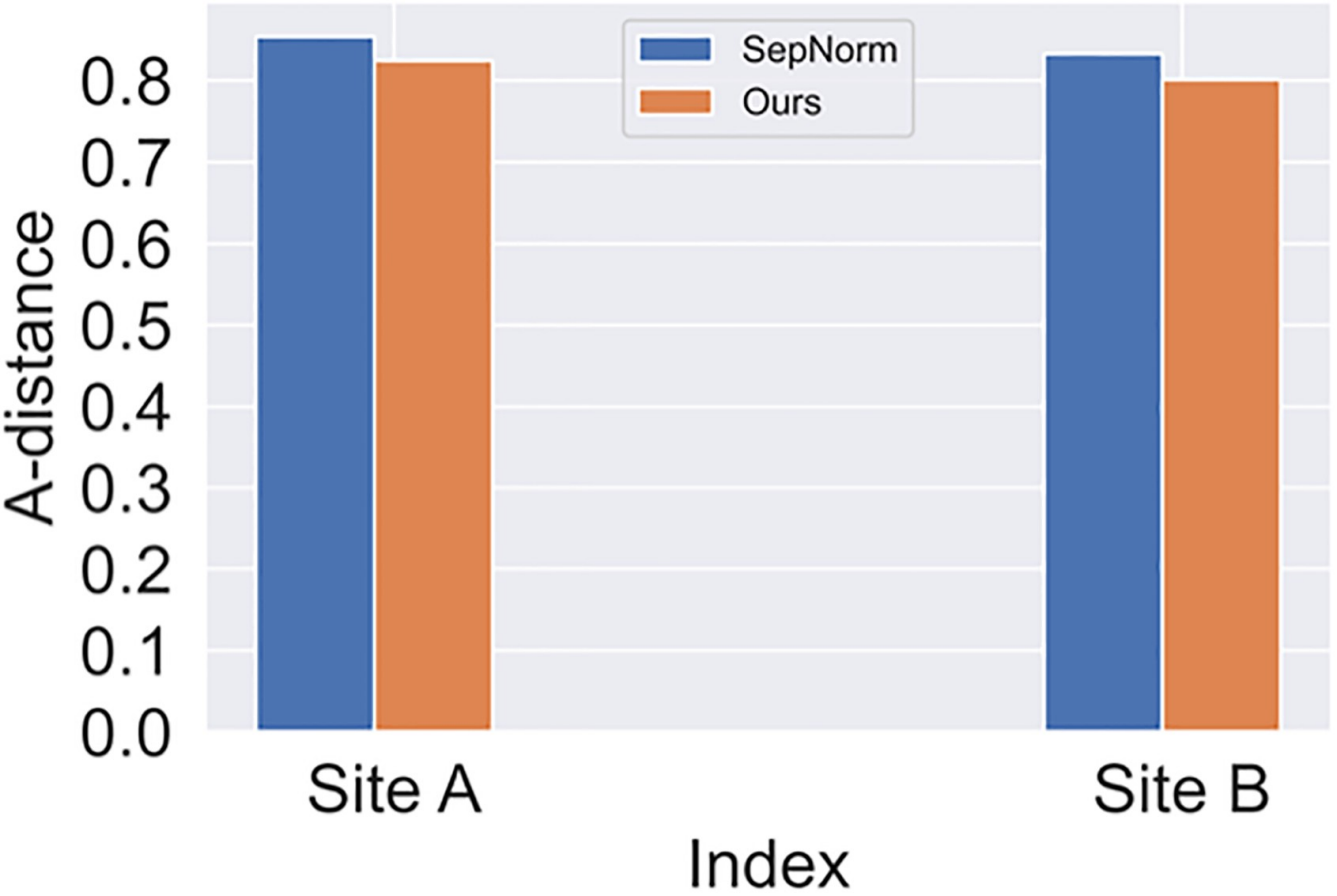
A -distance of SepNorm and ours.

We measure the difference between domains using the A-distance, which is a widely used metric for evaluating distribution divergence. The A-distance is defined as $A_{dis}=2\left(1-\epsilon \right)$, where *ϵ* represents the classification error of a trained classifier the distinguishes the source and target domains. A smaller A-distance indicates better alignment between the distributions. Our approach, illustrates in Fig 4, learns more invariant features, resulting in a reduction of the divergence between the source and target domains compared to other compared methods.

We conducted paired t-tests to analyze the significance of the improvements of our method over the *Joint*, *Single*, and *SepNorm* approaches. The detailed results are shown in Tables 3 and 4. This range corresponds to a 0.95 confidence interval, we observed that all paired t-tests presented a p-value smaller than 0.05, indicating statistically significant improvements of our method on both sites.

**Table 3:** The p-value with paired t-test of our method with Single, Joint and SepNorm learning schemes.

Methods	Single	Joint	SepNorm
Site A	0.002	0.007	0.023
Site B	0.004	0.005	0.009

**Table 4:** The p-value with paired t-test of our method with the state-of-the-art comparison methods.

Methods	Series-Adapter	Parallel-Adapter	MS-Net
Site A	0.009	0.012	0.021
Site B	1e-5	0.014	0.008

#### Comparison with state-of-the-art methods

We compared our method with recently proposed medical image analysis methods, comparing three different approaches. The first is **Series-Adapter** [36], which proposes multiple domain adapters for joint learning from multiple datasets and incorporates residual blocks into domain-adaptive layers to reduce the difference between source and target domain distributions. The second is **Parallel-Adapter** [37], which proposes parallel domain adaptation and inserts filter banks and domain-adaptive convolutional layers into residual blocks to reduce domain distribution differences. This method demonstrated state-of-the-art performance on 10 image classification datasets. **MS-Net** [7] also constructs a multi-source domain model, primarily utilizing auxiliary domain-specific branches to improve feature learning ability and employing online learning strategies to enhance the segmentation performance of MRI datasets.

The evaluation of joint learning methods is based on the Joint approach. Table 1 shows that the Series Adapter performs better than the Joint model in both Site A and Site B. However, its improvements are unevenly distributed across both sites and still fall short of the Single approach. On the other hand, the Parallel Adapter achieves more balanced improvements over the Joint model. These two approaches’ improvements over the Joint model suggest that domain-specific parameters in the domain adapter are useful in addressing the issue of data heterogeneity. The MS-Net outperforms the two domain-adaptive approaches, indicating the advantages of the knowledge transfer process in this framework. Notably, our method surpasses all three state-of-the-art joint learning methods in both sites, highlighting the effectiveness of our approach in leveraging more robust representations from diverse datasets.

## Conclusion

Our aim in this paper is to develop a highly accurate model for COVID-19 CT diagnosis by exploring the benefits of joint learning from heterogeneous datasets of different data sources. To achieve this, we propose a novel joint learning framework that utilizes a redesigned version of the recently proposed COVID-Net as a strong backbone. Our joint learning framework explicitly addresses the inter-site data heterogeneity by conducting separate feature normalization for each site. Additionally, we utilize a contrastive training objective to enhance the learning of domain-invariant semantic features that improve the identification performance on each dataset. The effectiveness and clinical significance of our approach are demonstrated through experiments conducted on two large-scale public datasets. However, we acknowledge that there is scope for further improvement and generalization of our approach. As future works, we aim to extend our model to a wider multi-site setting and employ transfer learning from other large-scale datasets to further enhance the diagnosis accuracy. We recognize the importance of improving the generalization capacity of our model to enhance its practical applicability. Overall, our proposed joint learning framework has shown promising results in improving the accuracy of COVID-19 CT diagnosis, and we believe that it has the potential to make a significant impact in the field of medical image analysis.

## Further discussion

While our method has shown promising performance as a preliminary study of multi-site learning with COVID-19 data, there are still limitations that need to be addressed. One of the limitations is that our method is currently limited to the two sites used in our paper, making it suboptimal for direct application to other unseen sites. This limits the potential for wider cross-site deployment. Additionally, due to computational resource constraints and development urgency, we were unable to pretrain our redesigned model on large-scale datasets such as ImageNet. However, previous works have demonstrated that fine-tuning transferred models can improve performance and speed up the training process. As a near future work, we are interested in exploring how to connect carefully redesigned network architectures with model transfer learning from large-scale datasets, to balance their respective benefits. Furthermore, we plan to extend our method to more sites with different environments to validate the generalization capability of AI models in the context of COVID-19 CT image diagnosis.
